# Cellular hnRNP A2/B1 interacts with the NP of influenza A virus and impacts viral replication

**DOI:** 10.1371/journal.pone.0188214

**Published:** 2017-11-16

**Authors:** Cheng-Kai Chang, Chi-Jene Chen, Chih-Ching Wu, Shiau-Wen Chen, Shin-Ru Shih, Rei-Lin Kuo

**Affiliations:** 1 Graduate Institute of Biomedical Sciences, College of Medicine, Chang Gung University, Taoyuan, Taiwan; 2 Department of Medical Laboratory Science and Biotechnology, China Medical University, Taichung, Taiwan; 3 Research Center for Emerging Viruses, China Medical University Hospital, Taichung, Taiwan; 4 Department of Medical Biotechnology and Laboratory Science, College of Medicine, Chang Gung University, Taoyuan, Taiwan; 5 Department of Otolaryngology-Head & Neck Surgery, Chang Gung Memorial Hospital, Linkou, Taoyuan, Taiwan; 6 Research Center for Emerging Viral Infections, College of Medicine, Chang Gung University, Taoyuan, Taiwan; 7 Clinical Virology Laboratory, Chang Gung Memorial Hospital, Linkou, Taoyuan, Taiwan; 8 Department of Pediatrics, Chang Gung Memorial Hospital, Linkou, Taoyuan, Taiwan; University of Hong Kong, HONG KONG

## Abstract

The viral ribonucleoprotein (vRNP) of influenza A virus is formed by virion RNA (vRNA), viral polymerase complex, and nucleoprotein (NP). The NP plays an important role in facilitating the replication and stabilization of viral RNA. To explore host factors that may be involved in the regulation of viral replication through interactions with NP, we conducted an immunoprecipitation experiment followed by mass spectrometry to identify NP-associated cellular proteins. Here, we demonstrate that NP can interact and colocalize with heterogeneous nuclear ribonucleoprotein (hnRNP) A2/B1 in mammalian cells and that the interaction may occur via direct binding to the glycine-rich domain (GRD) of hnRNP A2/B1. In addition, two residues in the tail loop of NP, F412 and R422, are required for the interaction of hnRNP A2/B1. Because the knockdown of hnRNP A2/B1 expression reduces viral RNP activity, hnRNP A2/B1 may act as a positive regulator in viral RNA synthesis of influenza A virus. More importantly, the findings in this research demonstrate that host proteins can regulate the replication of influenza A virus by interacting with NP.

## Introduction

Influenza A virus causes respiratory diseases in humans and leads to annual epidemics and pandemics worldwide. The virion of influenza A virus consists of eight segments of genomic RNA with negative polarity. Each of the virion RNA segments is associated with a viral polymerase complex and bound with viral nucleoprotein (NP) to form ribonucleoprotein (RNP). The viral polymerase complex comprises three subunits, PB1, PB2 and PA, and functions as a transcriptase and replicase to generate viral mRNA and virion RNA [1, 2].

NP from influenza A virus has been demonstrated to interact directly with the viral polymerase complex and enhance unprimed viral RNA replication [3, 4]. This interaction could lead to a conformation change in the polymerase complex to switch transcription activity to replication activity. In addition, NP could stabilize the replication intermediate of viral RNA, complementary RNA (cRNA), facilitating viral RNA replication [5]. However, host factors may also be involved in the regulation of influenza viral RNA synthesis through interactions with NP. For example, UAP56 was identified as a positive regulator of influenza viral RNA replication because it enhances the NP-RNA interaction [6]. In addition, it was determined recently that pre-mRNA processing factor 18 (Prp18) serves as a stimulatory factor, whereas Moloney leukemia virus 10 (MOV10) acts as a restriction factor for influenza viral RNA synthesis [7, 8]. Moreover, the regulation of NP activity in influenza viral RNA synthesis is also controlled by posttranslational modification. It has been found that the ubiquitination of NP at K184 might regulate viral genome replication [9]. Nevertheless, the sumoylation of NP might be involved in its trafficking and the facilitation of influenza virus growth without affecting viral polymerase activity [10].

Heterogeneous nuclear ribonucleoproteins (hnRNPs), which include more than 20 protein members, are RNA-binding proteins bound to pre-mRNA to form hnRNP particles in eukaryotic cells. These proteins typically contain an RNA recognition motif (RRM) and an arginine/glycine-rich domain (GRD) and play roles in several biological processes, such as transcription, RNA processing, and RNA trafficking and localization [11, 12]. The hnRNP A2/B1 gene encodes two isoforms of hnRNP, hnRNP A2 and B1. The B1 isoform is a splicing variant with extra 12 amino acids on the N-terminus of hnRNP A2 [13]. It has been shown that hnRNP A2 is involved in the transport of mRNA in cytoplasm by binding to a specific sequence named the hnRNP A2 response element (A2RE) [14, 15]. Furthermore, hnRNP A2 was copurified with pre-mRNA and considered to be a regulator that participates in alternative splicing [16, 17]. More recently, it was found that hnRNP A2/B1 binds to the *N*^*6*^-methyladenosine marks of primary miRNA transcripts and associates with the pri-miRNA microprocessor complex. This finding demonstrates that hnRNP A2/B1 is involved in pri-miRNA processing [18]. In addition to the functions in cellular RNA processing, hnRNP A2/B1 binds to A2RE on the genome of HIV-1 and modulates the trafficking and assembly of the viral genome [19, 20].

In the present study, we revealed, using co-immunoprecipitation and mass spectrometry (MS), that the NP encoded by influenza A virus could interact with cellular hnRNP A2/B1. The interaction of NP and hnRNP A2/B1 was found to be a direct protein–protein interaction between the GRD of hnRNP A2/B1. In addition, two residues, F412 and R422, on the tail loop of NP play a critical role in the interaction. Furthermore, we provide evidence suggesting that hnRNP A2/B1 could be a positive regulator of influenza A viral RNP activity and replication.

## Materials and methods

### Cells, viruses, and plasmids

A549, 293T, HeLa, and MDCK cells were cultivated with Dulbecco’s modified Eagle’s medium (DMEM) containing 10% fetal bovine serum. The cells were maintained in an incubator at 37°C with 5% CO_2_. Influenza A/WSN/33/H1N1 virus (WSN/H1N1) was amplified in MDCK cells and titrated by plaque formation assay with MDCK monolayers, as previously described [21]. The open reading frames (ORFs) of NP encoded by influenza A TW/141/2002/H1N1 (141/H1N1) and WSN/H1N1 were cloned into the expression vector pFLAG-CMV5.1 (Sigma-Aldrich, St. Louis, MO, USA). The cDNA of NP encoded by an avian influenza A H6N1 virus was provided by Dr. Ching-Ho Wang at the School of Veterinary Medicine, National Taiwan University, and sub-cloned into the pFLAG-CMV5.1 vector. Each of the plasmids expressing a single amino acid change of NP was generated by PCR-based site-directed mutagenesis and constructed in the pFLAG-CMV5.1 vector. The tail loop-deletion (Δ402–429) of NP ORF was amplified from a plasmid containing Δ402–429 NP (provided by Dr. Yizhi Jane Tao at Rice University) and inserted into the pFLAG-CMV5.1 vector. To construct the HA-tagged hnRNP A2 expression plasmid, the ORF of hnRNP A2 was amplified from the cDNA of A549 cells and cloned into the pCMV-HA vector (Clontech Laboratories, Mountain View, CA, USA). For purification of proteins in vitro, the ORFs of wild type NP were sub-cloned into the pET30a(+) vector (Novagen/EMD Millipore, Billerica, MA), and the ORFs corresponding to the RRM1, RRM2, and GRD regions of hnRNP A2 were constructed in the pGEX-5X-1 vector (GE Healthcare Life Sciences, Marlborough, MA, USA), which contains a glutathione S-transferase (GST) sequence.

### Immunoprecipitation and immunoblotting

The 293T cells that were transfected with a plasmid expressing FLAG-tagged NP (or co-transfected with the indicated NP construct and HA-tagged hnRNP A2 expression plasmid) were washed with PBS and then lysed in a lysis buffer containing 50 mM Tris-HCL (pH 7.4), 150 mM NaCl, 1 mM EDTA and 1% Triton X-100. After centrifugation at 12,000 X g for 30 minutes, the supernatants were collected and mixed with ANTI-FLAG M2 affinity gel (Sigma-Aldrich). The mixture was rotated at 4°C for 2 h, and the affinity gel was then collected after centrifugation at 8,000 X g for 30 seconds. The affinity gel was thrice washed with a buffer containing 50 mM Tris-HCl (pH 7.4) and 150 mM NaCl. The FLAG-tagged NP and the associated proteins on the affinity gel were eluted with 3X FLAG peptide (Sigma-Aldrich) in wash buffer. The eluted proteins were then separated on SDS-polyacrylamide gels and transferred to PVDF membranes. After blocking with 5% skim milk, the membranes were reacted with anti-FLAG (1:1000 dilution, Sigma-Aldrich), anti-HA (1:1000 dilution, Sigma-Aldrich), anti-hnRNP A1 (1:1000 dilution, Santa Cruz Biotechnology), anti-hnRNP A2/B1 (1:1000 dilution, Sigma-Aldrich) or anti-β-actin antibodies (1:2000 dilution, EMD Millipore) and then detected with an HRP-conjugated anti-mouse or anti-rabbit IgG antibody (1:5000 dilution, GE Healthcare Life Sciences).

### In-gel digestion of selected protein band

Proteins were separated on a 10% SDS gel and then subjected to silver staining. Protein bands were excised, destained, and subjected to in-gel digestion with trypsin, as previously described [22, 23]. Briefly, the protein band was destained with 1% potassium ferricyanide and 1.6% sodium thiosulfate (Sigma-Aldrich). Then, the proteins were reduced with 25 mM NH_4_HCO_3_ (Sigma-Aldrich) containing 10 mM dithiothreitol (Biosynth AG, Switzerland) at 60°C for 30 min and alkylated with 25 mM NH_4_HCO_3_ containing 55 mM iodoacetamide (Amersham Biosciences, Buckinghamshire, UK) at room temperature for 30 min. After reduction and alkylation, the proteins were digested with sequencing-grade modified porcine trypsin (20 μg/mL; Promega, Madison, WI, USA) at 37°C for 16 h.

### Mass spectrometry (MS) analysis and database search

The in-gel tryptic peptides were extracted with acetonitrile (Mallinckrodt Baker, NJ, USA) containing 0.5% trifluoroacetic acid (Sigma-Aldrich) and then loaded onto an MTP AnchorChip^TM^ 600/384 TF (Bruker Daltonics, Bremen, Germany). Peptide mass was determined using an Ultraflex^TM^ MALDI-TOF mass spectrometer (Bruker Daltonics) controlled by the FlexControl 2.2 software package (Bruker Daltonics). Peptide mass fingerprints (PMFs) were acquired in reflectron mode (26.7 kV accelerating voltage) with 300 laser shots per spectrum.

Peptide masses were annotated using FlexAnalysis software (version 2.2, Bruker Daltonics); the settings were signal-to-noise threshold = 2; peak detection algorithm = SNAP; peak width = 0.75 *m/z*; maximal number of peaks = 200; and quality factor threshold = 50. The acquired masses were internally calibrated to a mass accuracy within 50 ppm, using a peptide mixture of ACTH (*m/z* 2465.19), human angiotensin II (*m/z* 1046.54), and bovine serum albumin (*m/z* 927.49). Annotated and calibrated masses were searched with the Mascot search engine (version 2.1, Matrix Science, MA, USA) in BioTools 2.2 software (Bruker Daltonics) against the Swiss-Prot human sequence database (released Apr 16, 2014, selected for *Homo sapiens*, 20,265 entries) of the European Bioinformatics Institute. The mass tolerance of the ions was set to 50 ppm, with trypsin as the digestion enzyme. Up to one missed cleavage was allowed, and searches were performed with the parameters of variable oxidation on methionine (+15.99 Da) and fixed carbamidomethylation on cysteine (+57 Da). Protein with statistically significant search scores (greater than 95% confidence interval, equivalent to Mascot expected p value < 0.05) were treated as positive identifications. MS spectra with multiple matches were manually inspected to ensure the correct PMF assignment.

### Immunofluorescence and confocal microscopy

A549 cells grown on coverslips were infected with influenza A WSN virus at a multiplicity of infection (MOI) 2. At the indicated time points, the cells were fixed with 4% paraformaldehyde. After treating the fixed cells with 0.3% Triton X-100, the cells were subjected to immunofluorescence staining, as previously described [24]. Briefly, the fixed cells were blocked with 5% bovine serum albumin and incubated with monoclonal anti-hnRNP A2/B1 (Sigma-Aldrich) and polyclonal anti-NP antibodies, which were generated in house. The fluorescence-conjugated antibodies (Alexa Fluor 488-conjugated anti-mouse and Alexa Fluor 555-conjugated anti-rabbit; Life Technologies, Carlsbad, CA, USA) were applied to detect the primary antibodies. Nuclei were stained with 4’ 6-diamidino-2-phenylindole (DAPI). The localization of the indicated proteins and nuclei was then observed on a ZEISS LSM510 META confocal microscope.

### Protein purification and glutathione Sepharose affinity selection

The 6X His-tagged NP was expressed and purified, as described previously [25]. The GST-fused hnRNP A2 constructs were transformed into *Escherichia coli* BL21 cells, and they were expressed under induction by isopropyl-β-D-thiogalactopyranoside. The *E*. *coli* cells were harvested by centrifugation and then disrupted by sonication. The supernatants of the cell lysates were subjected to a glutathione Sepharose 4B affinity column (GE Healthcare Life Sciences) to purify the full-length, and RRM1, RRM2, and GRD regions of GST-fused hnRNP A2. The purified 6X His-tagged NP was incubated with the purified full-length or different regions of GST-fused hnRNP A2 at 4°C for 60 min. A glutathione Sepharose 4B affinity column was used to pull down the GST-tagged proteins. The 6X His-tagged NP associated with the GST-fused proteins was detected by SDS-PAGE and immunoblotting.

### siRNA transfection and detection of viral RNA synthesis

HeLa cells were transfected with siRNA against hnRNP A2/B1 (siRNA duplex synthesized by Sigma-Aldrich. #1: 5’-CAGAAGAAAGUUUGAGGAACUACUA- 3’ & 5’-UAGUAGUUCCUCAAACUUUCUUCUG-3’; #2: 5’-GAAGCUGUUUGUUGGCGGAAUUAAA-3’ & 5’-UUUAAUUCCGCCAACAAACAGCUUC-3’), siRNA against hnRNP A1 (5’-GGAAGAGUUGUGGAACCAA-3’ & 5’-UUGGUUCCACAACUCUUCC-3’, synthesized by Sigma-Aldrich), or control siRNA (5’-UUCUCCGAACGUGUCACGUTT-3’ & 5’-ACGUGACACGUUCGGAGAATT-3’, synthesized by Sigma-Aldrich) with Lipofectamine 2000 (Invitrogen, Carlsbad, CA, USA) for 24 h and then co-transfected with plasmids that expressed PB1, PB2, PA, and NP from influenza A/WSN/H1N1 virus and a polymerase I promoter-driven plasmid expressing a negative-sensed ORF of firefly luciferase bearing 5’ and 3’ untranslated regions of NS vRNA. A Renilla luciferase reporter under polymerase II promoter control was also co-transfected to monitor transfection efficiency. At 24 h post-transfection, the lysates of the transfected cells were collected and the relative RNP activity was measured using a Dual-Luciferase Reporter Assay (Promega), as described previously [26]. The total RNA of the transfected cells was extracted, treated with DNase I (Promega), and subjected to reverse transcription (RT) and real-time PCR to detect the model vRNA and the mRNA of the reporter (firefly luciferase). The design of the primers for the RT real-time PCR were designed as described in a previous publication [4] and are summarized in S1 Table.

### Determination of influenza A virus replication

A549 cells were transfected with siRNA against hnRNP A2/B1 for 24 h and then reseeded onto cell culture dishes for 24 h. The transfected cells were infected with influenza A WSN virus at MOI of 0.001. The supernatants and lysates of the infected cells were collected at the indicated time post-infection. The infectious viruses in the supernatant were titrated by plaque formation assay with monolayers of MDCK cells, as described previously [21].

## Results

### Influenza A virus nucleoprotein interacts with cellular hnRNP A2/B1 protein

**Fig 1:**
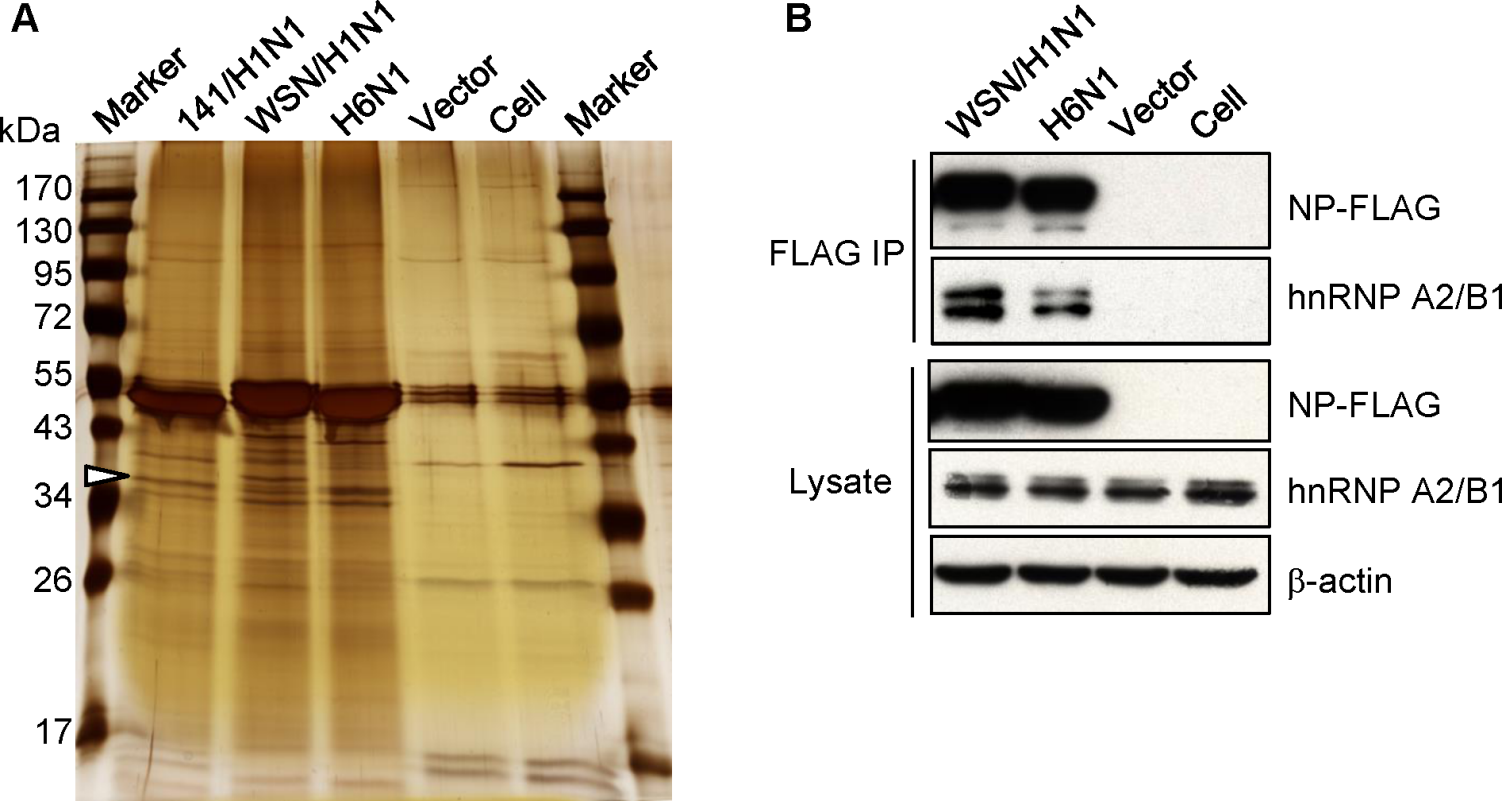
Identification of the interaction of influenza NP and hnRNP A2/B1. (A) 293T cells were transfected with a plasmid that expresses FLAG-tagged NP encoded by influenza A 141/H1N1, WSN/H1N1, an H6N1 virus, or an empty vector. The lysates of transfected cells were subjected to anti-FLAG immunoprecipitation. The precipitates were separated by SDS-PAGE, and the gels were then subjected to silver staining. The indicated band was excised, digested, and subjected to MS for protein identification, as described in the materials and methods. (B) Lysates of 293T cells transfected with the indicated FLAG-tagged NP expressing plasmids were collected and subjected to anti-FLAG immunoprecipitation. The proteins in the lysates and precipitates were analyzed by SDS-PAGE and immunoblotting with anti-FLAG, anti-hnRNP A2/B1 and anti-β actin antibodies.

To explore novel functions of the NP protein encoded by influenza A virus in host cells, lysates of 293T cells transfected with plasmids expressing the FLAG-tagged NP of influenza 141/H1N1, WSN/H1N1, or an H6N1 virus were subjected to anti-FLAG immunoprecipitation and MS to search for cellular proteins that could associate with the NP protein. As shown in Fig 1A, the precipitates were separated by SDS-PAGE and visualized by silver staining. Compared to the vector control, we found that bands with sizes of approximately 36 kDa, as indicated in the figure, only appeared in the precipitates of NP-expressing lysates. One of the bands was excised and identified as hnRNP A2/B1 by MS. To validate the interaction of NP and hnRNP A2/B1, the precipitates were also analyzed by immunoblotting with an anti-hnRNP A2/B1 antibody (Fig 1B). The results demonstrated that influenza A NP protein may associate with cellular hnRNP A2/B1 protein. These results indicate that NP may interact with hnRNP A2/B1 in host cells.

**Fig 2:**
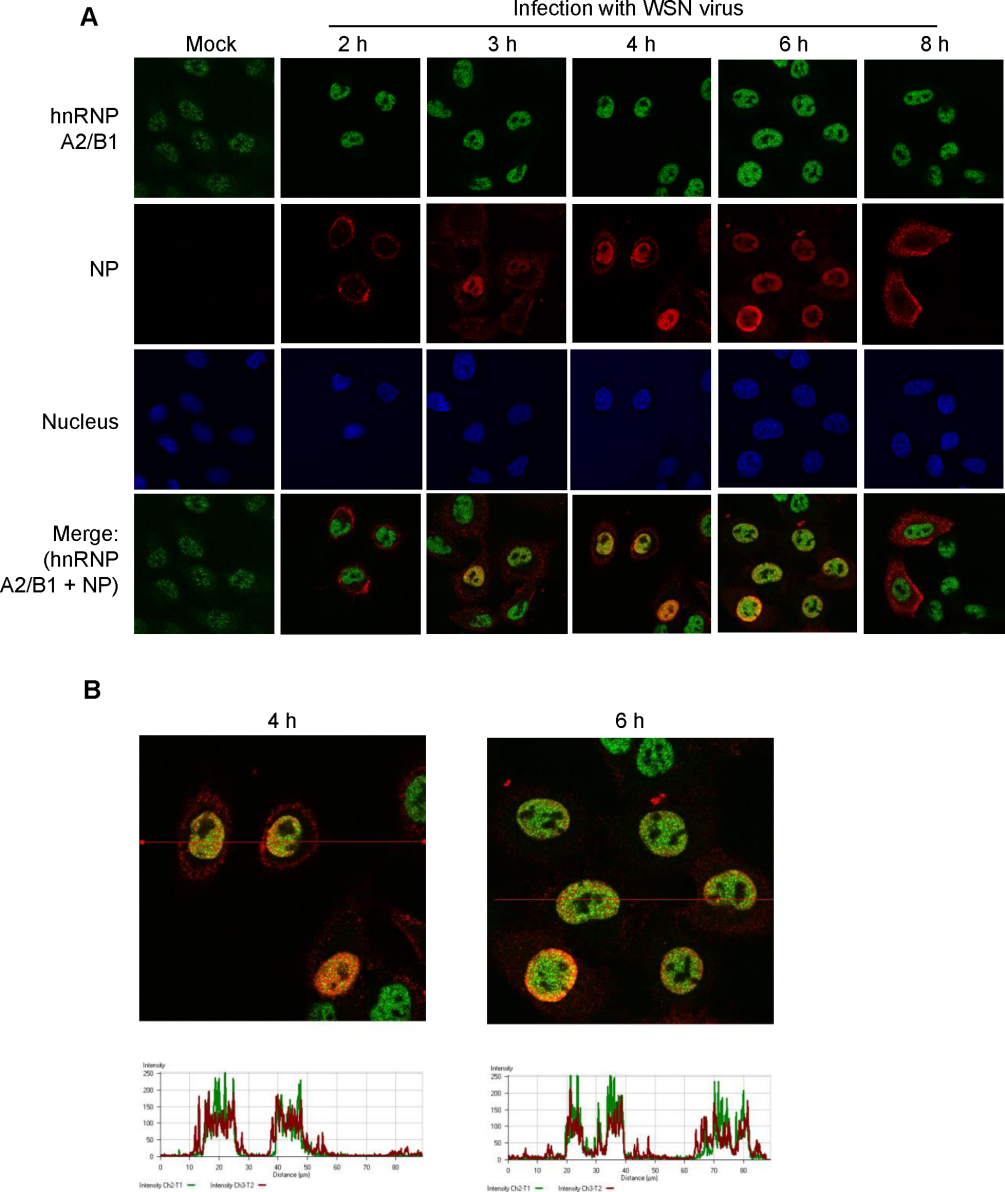
The localization of hnRNP A2/B1 and the NP of influenza A virus during infection. (A) A549 cells were infected or mock-infected with the influenza A WSN/H1N1 virus. At the indicated time points, the localization of viral NP and hnRNP A2/B1 in cells was examined by immunofluorescence staining with anti-NP and anti-hnRNP A2/B1 antibodies. (B) The fluorescence signal of the indicated lines in the images of immunofluorescence staining at 4 and 6 h post-infection were analyzed by LSM Image Browser.

To determine whether influenza NP can be colocalized with hnRNP A2/B1 during infection with the influenza A virus, A549 cells were infected with the WSN/H1N1 strain of influenza A, and the localization of NP and hnRNP A2/B1 was observed by confocal microscopy at 2, 3, 4, 6, and 8 h post-infection. As shown in Fig 2A, hnRNP A2/B1 was localized in the nucleus during infection, whereas NP was transported from the cytoplasm to the nucleus and was mainly localized in the nucleus at 3, 4 and 6 h after infection. The immunofluorescence demonstrated that NP and hnRNP A2/B1 could be colocalized after NP was transported to the nucleus. We then quantitated the fluorescence intensity of the images obtained at 4 and 6 h post-infection using LSM Image Browser (Fig 2B). The results demonstrated that NP and hnRNP A2/B1 were colocalized during influenza A viral infection.

### Influenza A NP binds to the glycine-rich domain of hnRNP A2/B1 in vitro

**Fig 3:**
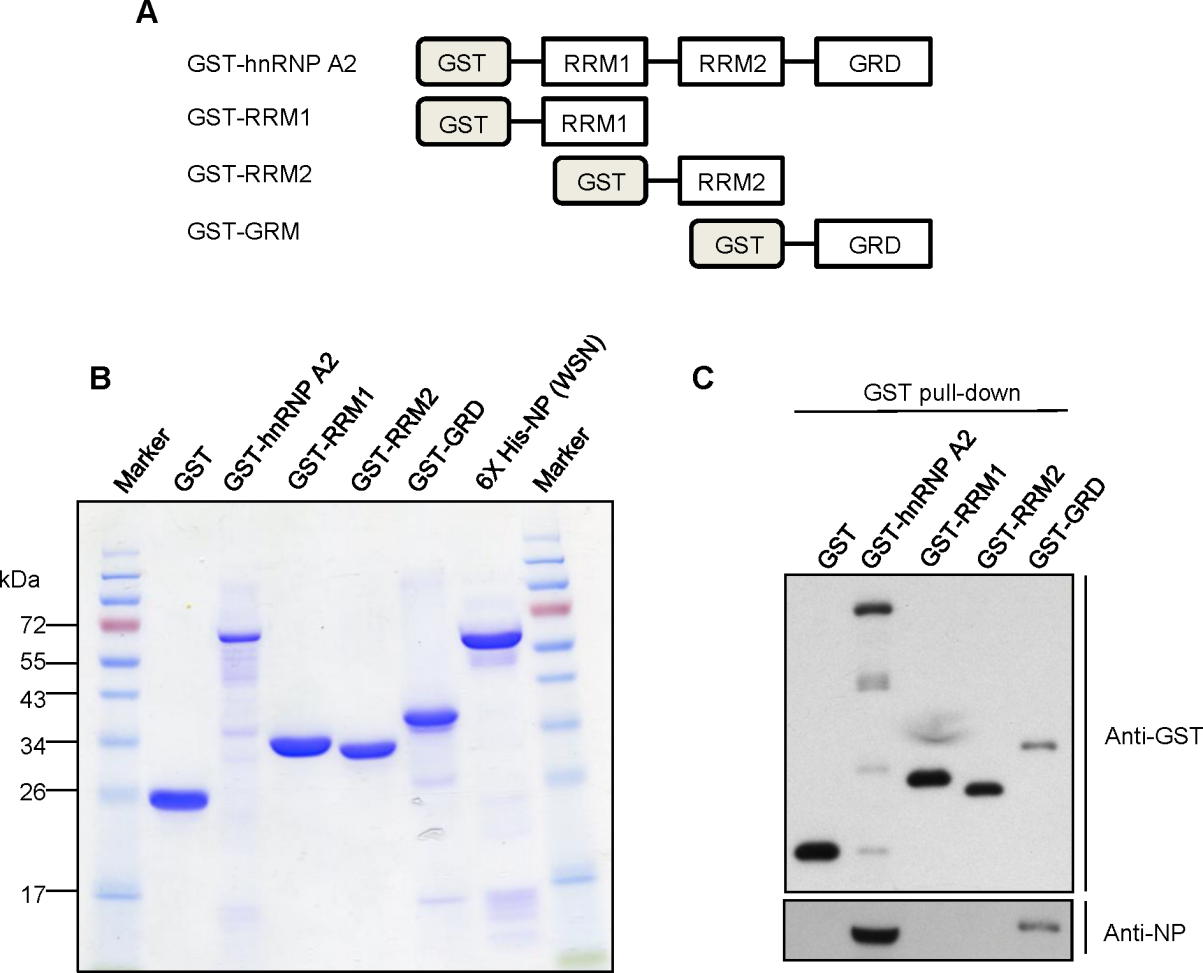
The binding of NP of influenza A virus and the GRD of hnRNP A2 in vitro. (A) Diagram of GST-fused hnRNP A2 recombinant proteins used in the in vitro binding assay. (B) Purified GST-fused hnRNP A2-related and 6X His-tagged NP were examined by SDS-PAGE and Coomassie blue staining. (C) The purified 6X His-tagged NP was incubated with the indicated GST-fused hnRNP A2 recombinant proteins. A glutathione affinity column was used to pull down the GST-tagged proteins. The proteins in the pull-down assay were analyzed by SDS-PAGE and immunoblotting with anti-GST and anti-NP antibodies.

To evaluate whether NP could directly bind to hnRNP A2/B1 without mediation by other factors or RNA, we expressed and purified the following recombinant proteins: His-tagged NP; GST-fused full-length; and the RRM1, RRM2, and GRD regions of hnRNP A2/B1 (Fig 3B). Then, we performed GST pull-down assays after incubating mixtures of the recombinant proteins. We found that His-tagged NP could be pulled down by the full-length or GRD region of hnRNP A2/B1 (Fig 3C). These results clearly demonstrated that NP could directly bind to the GRD of hnRNP A2/B1. Taken together, these results indicate that NP encoded by influenza A virus interacts with cellular hnRNP A2/B1 via a direct protein–protein interaction.

### Residues F412 and R422 in the tail loop of NP are crucial for the interaction with hnRNP A2/B1

**Fig 4:**
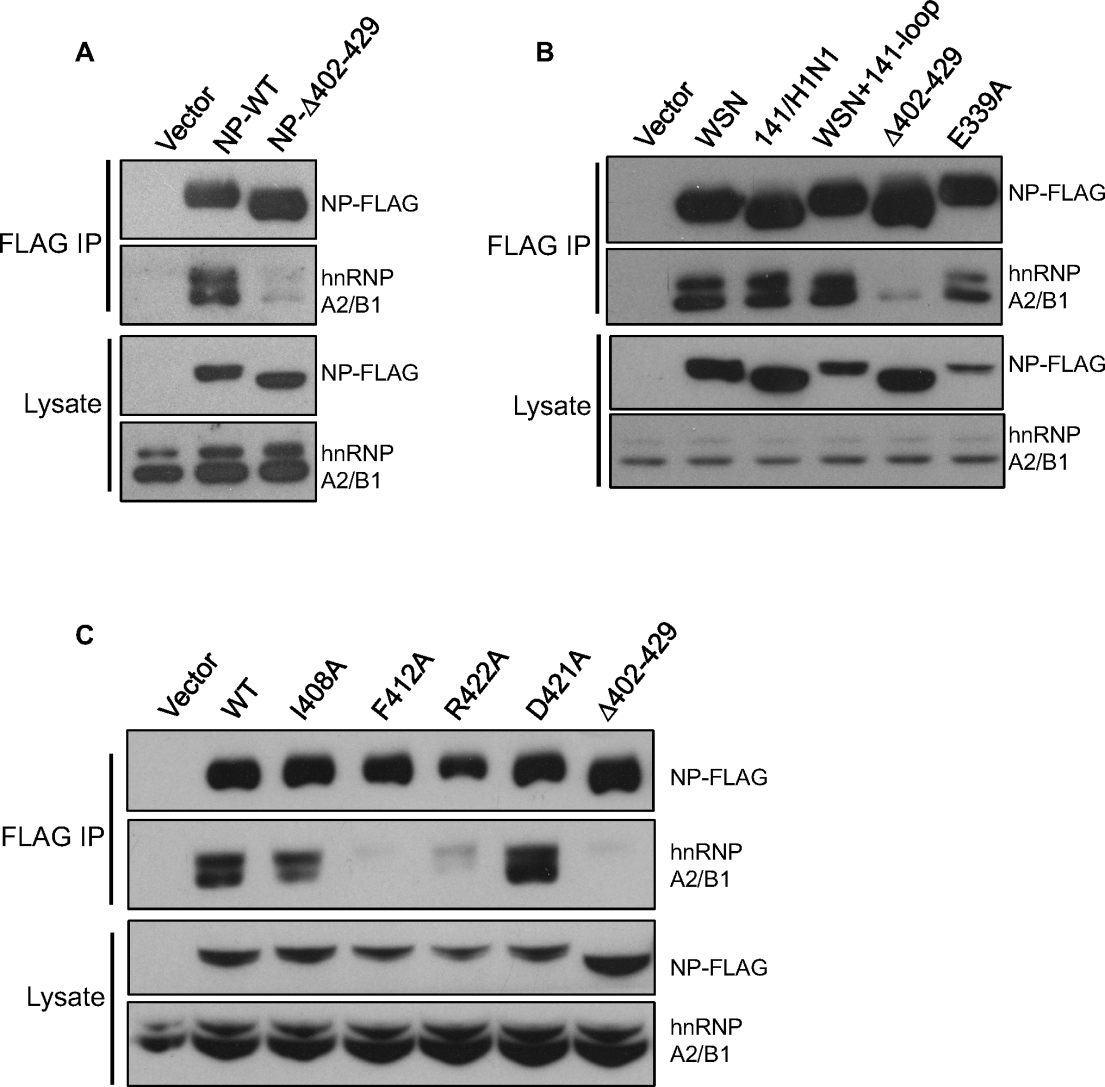
Identification of the F412 and R422 residues in the tail loop of NP for interacting hnRNPA2/B1. (A) 293T cells were transfected with a plasmid that expresses either wild type or tail loop-deleted (Δ402–428) NP of WSN virus for 24 h. Lysates of the transfected cells were subjected to anti-FLAG immunoprecipitation. The precipitates were analyzed by SDS-PAGE and immunoblotted with anti-NP and anti-hnRNP A2/B1 antibodies. (B and C) Lysates of 293T cells transfected with a plasmid expressing wild type NP of WSN, 141/H1N1 virus, or the indicated mutants were subjected to anti-FLAG immunoprecipitation. As mentioned, the precipitates were analyzed by immunoblotting.

Previous studies have shown that the tail loop (containing residues 402–429) of the NP protein plays important roles in oligomerization and interactions with other cellular factors [26–28]. We further determined whether this region is crucial for the interaction of NP and hnRNP A2/B1. The 293T cells were transfected with plasmids expressing either the wild type or tail loop-deletion (denoted Δ402–429) of FLAG-tagged NP encoded by the WSN virus. At 48 h post-transfection, lysates of the transfected cells were collected and subjected to anti-FLAG immunoprecipitation. Compared to the WT NP, the Δ402–429 NP had a dramatically reduced ability to interact with hnRNP A2/B1 (Fig 4A). Because four residues were varied in the tail loop of NP encoded by 141/H1N1 virus, we further determined the interaction of hnRNP A2/B1 with the NP of 141/H1N1. As shown in Fig 4B, the interaction between hnRNP A2/B1 and the NP of 141/H1N1 or the mutated WSN NP containing the four-residue change in the tail loop (S403A, I408T, R422K, and P423T; denoted WSN+141-loop) was similar to the interaction with the wild type NP of the WSN virus. Additionally, as mentioned earlier, the tail loop is crucial for oligomerization. We therefore investigated whether the oligomerization of NP is required for the interaction with hnRNP A2/B1. Immunoprecipitation of the E339A NP mutant, which is also an important residue for the formation of NP oligomers, was performed with the lysates of transfected cells. The results demonstrated that the E339A mutation in NP did not dramatically change the NP-hnRNP A2/B1 interaction (Fig 4B). These findings suggest that oligomerization of NP may not be required for the interaction with hnRNP A2/B1.

To further identify the residues that are required for the interaction, we examined the interaction of hnRNP A2/B1 and NP with single residue mutation: I408A, F412A, R422A, or D421A, in the tail loop. As shown in Fig 4C, the F412A and R422A mutants were unable to interact with hnRNP A2/B1 in transfected cells as the Δ402–429 mutant. These results demonstrated that the residues in the tail loop (F412 and R422) of the NP protein are crucial for interactions with hnRNP A2/B1.

### Knockdown of hnRNP A2/B1 expression reduces the synthesis of influenza viral RNA

**Fig 5:**
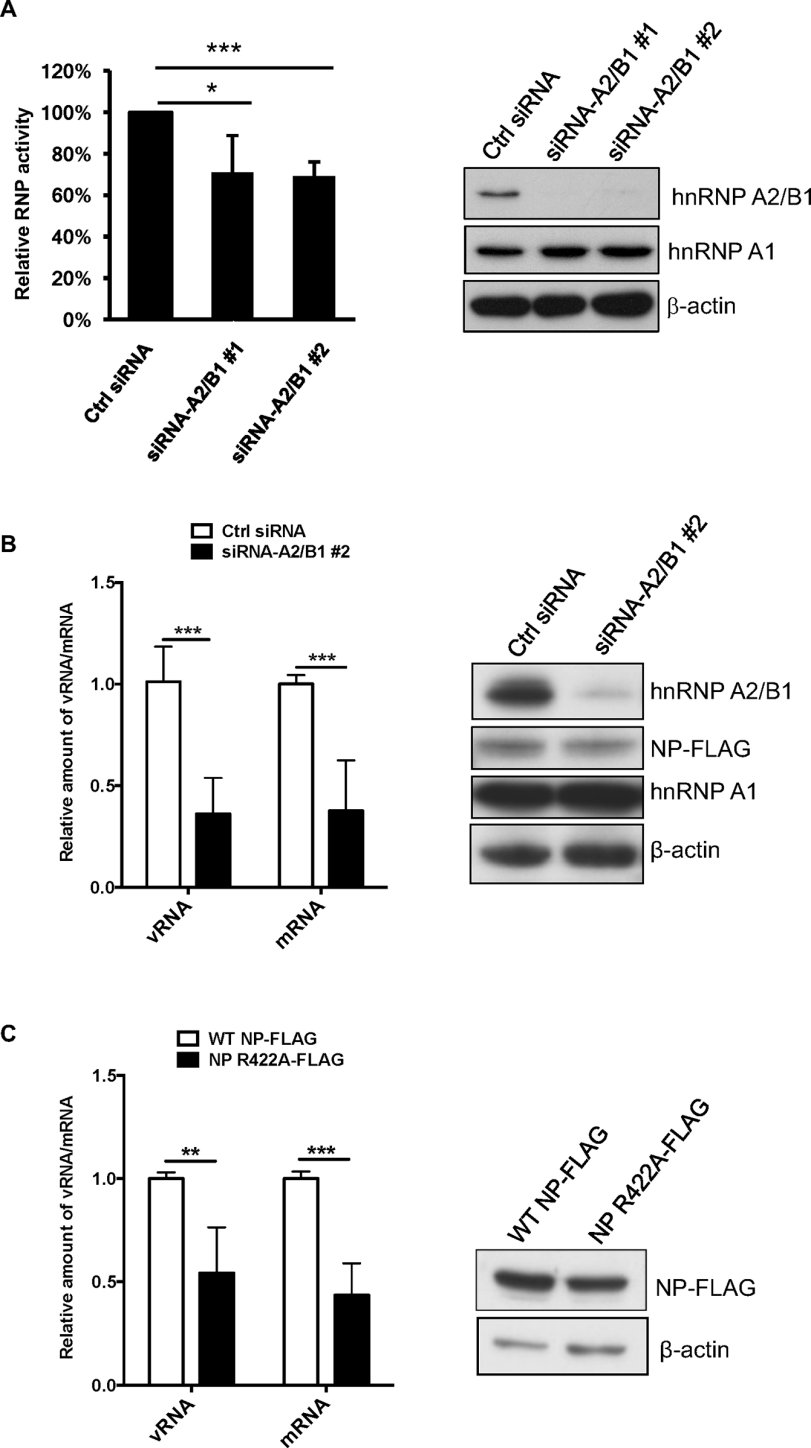
hnRNP A2/B1 is involved in influenza viral RNP activity. (A) HeLa cells were transfected with siRNA against hnRNP A2/B1 (either #1 or #2 siRNA) and then co-transfected with plasmids that express NP, PA, PB1, PB2, renilla luciferase, and a reporter vRNA containing an antisense of luciferase ORF flanked with noncoding regions of the NS segment. At 24 h post-transfection, lysates from the transfected cells were collected and analyzed with the Dual-Luciferase Reporter Assay and by immunoblotting with anti-hnRNP A2/B1 and anti-β actin antibodies. (B) Total RNA of the co-transfected cells was extracted and subjected to RT real-time PCR with the primers described in S1 Table to examine vRNA and mRNA production of firefly luciferase gene under normalizing with the mRNA of renilla luciferase gene. The protein expression of FLAG-tagged NP, hnRNP A2/B1, hnRNP A1, and β-actin were analyzed by immunoblotting with indicated antibodies. (C) HeLa cells were co-transfected with plasmids that express PA, PB1, PB2, the vRNA-like firefly luciferase RNA, and either wild type or R422A-mutated NP. At 24 h posttransfection, vRNA and mRNA of the reporter were examined as described previously. *P < 0.05; ** *P* < 0.01; *** *P* < 0.005.

Because NP is crucial in facilitating influenza viral polymerase in viral genome replication, our previous results prompted us to investigate the role of hnRNP A2/B1 in regulating the RNP activity of influenza A virus. To this end, influenza viral RNA synthesis was analyzed in HeLa cells with knockdown of hnRNP A2/B1 expression by siRNA transfection. The hnRNP A2/B1 knockdown cells were then co-transfected with expression plasmids that express NP, PA, PB1, PB2, and a vRNA-like reporter containing an antisense firefly luciferase ORF flanked by noncoding regions of the NS segment. At 24 h post-transfection, total RNA of the transfected cells was collected and subjected to RT and real-time PCR to determine the levels of vRNA and mRNA transcribed by the RNP complex, which represents the activity of the influenza polymerase complex (i.e., viral RNP). The results demonstrated that knockdown of hnRNP A2/B1 expression could reduce the RNP activity determined by the luciferase activity assay (Fig 5A), as well as the synthesis of vRNA and reporter mRNA (Fig 5B). Collectively, our results revealed that the NP-interacting cellular protein hnRNP A2/B1 may have a positive role in regulating influenza viral RNA synthesis. In addition, we determined the function of the NP R422A mutant, which does not bind to hnRNP A2/B1, in facilitating replication and transcription of viral RNAs. As shown in Fig 5C, the levels of virus-like mRNA and vRNA in the cells co-transfected with the R422A NP mutant was decreased. This result suggested that the interaction of influenza NP and hnRNP A2/B1 may promote the synthesis of influenza viral mRNA.

Because hnRNP A1 is highly homologous to hnRNP A2/B1, we also examined whether hnRNP A1 could interact with influenza NP and have a regulatory function in viral RNA synthesis. The lysates of 293T cells transfected with a FLAG-tagged NP expressing plasmid were subjected to anti-FLAG immunoprecipitation. Interestingly, hnRNP A1 was detected in the precipitates by immunoblotting with an anti-hnRNP A1 antibody (S1A Fig). We further determined whether hnRNP A1 is involved in regulating influenza RNP activity. HeLa cells were transfected with siRNA against hnRNP A1, and were then co-transfected with plasmids expressing influenza polymerase components, NP, and the vRNA-like firefly luciferase reporter. Interestingly, the activity of the firefly luciferase reporter was decreased in the cells knocked down with hnRNP A1 (S1B Fig). This result indicated that, like hnRNP A2/B1, hnRNP A1 may play a positive role in influenza viral RNA synthesis.

### hnRNP A2/B1 is involved in influenza virus replication

**Fig 6:**
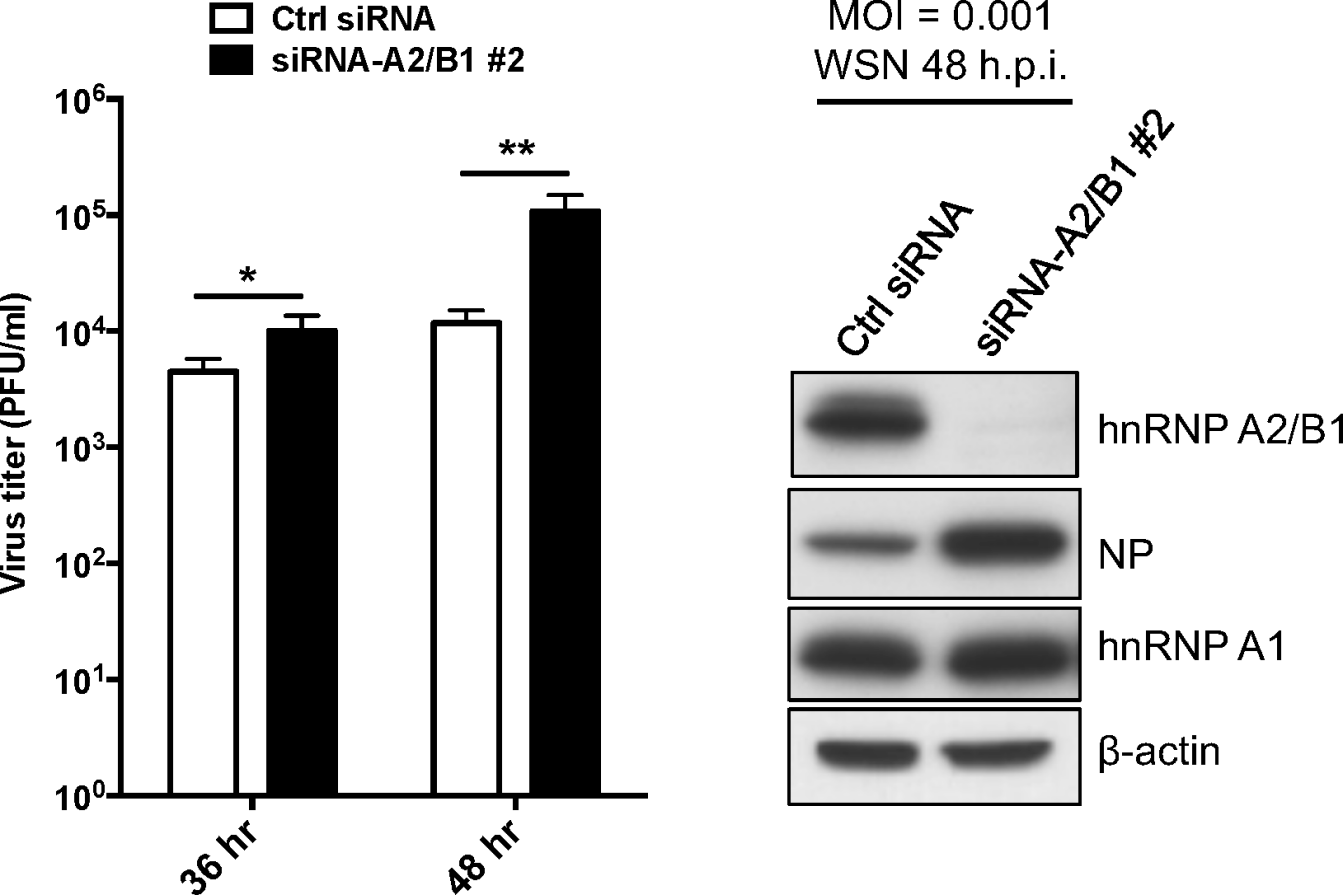
hnRNP A2/B1 is a positive regulator of influenza A viral replication. A549 cells were transfected with siRNA against hnRNP A2/B1 (#2 siRNA) or control siRNA for 24 h, and then infected with influenza A WSN virus at MOI of 0.001. At the indicated time points, the supernatants of the infected cells were collected and titrated via a plaque formation assay. The expression of hnRNP A2/B1 was monitored by immunoblotting with anti-hnRNP A2/B1 and anti-β actin antibodies. *P < 0.05; ** *P* < 0.01.

Previous results have demonstrated that the interaction of influenza NP and host hnRNP A2/B1 impacts viral RNP activity. We then investigated the role of hnRNP A2/B1 in influenza virus replication. A549 cells were transfected with siRNA against hnRNP A2/B1 expression for 24 h and then reseeded for 24 h. The transfectants were infected with influenza A/WSN/H1N1 virus at an MOI of 0.001. At 36 and 48 h post-infection, the supernatants and lysates of the infected cells were collected to determine virus titers and hnRNP A2/B1 expression. The result showed that knockdown of hnRNP A2/B1 significantly increased the replication of influenza A WSN virus in A549 cells (Fig 6). These data suggest that hnRNP A2/B1, which binds to influenza NP via its GRD, also participates in the replication of influenza A virus.

## Discussion

The NP encoded by influenza A viruses plays a critical role in viral RNA replication. Previous studies have revealed that NP can interact with the polymerase complex of the influenza A virus to facilitate viral RNA synthesis [3, 4]. In addition, host proteins may interact with NP to regulate influenza virus RNA synthesis [28]. To explore the functions of influenza A NP in viral replication, we conducted immunoprecipitation and MS to identify host factors that could interact with the NP of influenza A virus. In the present study, we found that human hnRNP A2/B1 could interact and colocalize with NP encoded by influenza A virus in cells. We further demonstrated that NP directly bound to the GRD of hnRNP A2/B1 in vitro and that the tail loop of NP, residues 402–429, was required for the interaction of NP and hnRNP A2/B1. Since our results also demonstrated that the knockdown of hnRNP A2/B1 expression reduced the viral RNA synthesis in mammalian cells, hnRNP A2/B1 is considered to be a positive regulator of the activity of influenza A viral RNP. In agreement with previous research, this finding implies that host proteins may regulate the replication of influenza A virus by interacting with the nucleoprotein.

NP forms oligomers, and the tail loop of NP is crucial for the inter-subunit interactions for oligomerization [27]. Although mutations on two residues in the tail loop, F412A and R422A, abolished the interaction of NP and hnRNP A2/B1, our results also demonstrated that the E339A mutation, which may also affect the oligomerization of NP, did not change the interaction of hnRNP A2/B1. Therefore, the oligomerization of NP might not be necessary for the interaction of hnRNP A2/B1. Nevertheless, with regard to NP oligomer formation, additional studies are required to examine whether hnRNP A2/B1 could impact inter-subunit interactions or the oligomerization of NP.

Because NP is involved in influenza viral RNA synthesis, hnRNP A2/B1 might contribute to viral RNA synthesis by regulating the activity of the viral polymerase complex. We used a mini genome reporter assay to determine the ability of influenza polymerase to perform viral RNA synthesis, and we observed a reduced level of the viral-model RNAs derived from the reporter in hnRNP A2/B1 knockdown cells. Consistently, we found that a mutation at R422 of the NP, which was demonstrated to be a key residue for the interaction with hnRNP A2/B1, decreased the ability to support influenza polymerase activity. This result was consistent with previous research that examined the mutation in the NP of influenza H5N1 virus [29]. Nevertheless, F412 of NP was also found to be an important position for interaction with NF90, which is a negative regulator of influenza A polymerase activity [28]. Therefore, understanding the cooperative roles of hnRNP A2/B1 and NF90 in regulating the activity of polymerase complex of influenza A virus requires further evaluation.

hnRNP A2/B1 may be involved in pre-mRNA processing in cells [11]. It remains unclear whether influenza NP could interfere with host pre-mRNA splicing via interactions with hnRNP A2/B1 to support viral RNA synthesis. Additionally, we have not ruled out the possibility that hnRNP A2/B1 may facilitate influenza A viral RNA replication by regulating some other host processes in infected cells. In addition, because hnRNP A2/B1 could function in the trafficking of mRNA and miRNA [15, 20, 30] and modulate N^6^-methyladenosine-dependent miRNA maturation processing [30], the interaction of influenza NP and hnRNP A2/B1 might also be linked to the generation of cellular miRNAs that regulate the expression of other cellular factors to facilitate influenza viral RNA synthesis.

As previously reported, hnRNP A2/B1 could interact with the NS1 protein of influenza A PR/8/H1N1 virus, and the reduction of hnRNP A2/B1 expression could contribute to the replication of the virus as a result of increasing levels of NS1 mRNA in the cytosol [31]. However, although we found that hnRNP A2/B1 interacts with NP of influenza A virus, and that the viral RNP activity was reduced in cells in which hnRNP A2/B1 expression had been knocked down, our data showed that knockdown of hnRNP A2/B1 increased the replication of influenza A WSN virus in A549 cells as the previous report [31]. Therefore, hnRNP A2/B1 has dual functions during influenza A virus infection. The protein binds with influenza NP and works as a positive regulator in the synthesis of the influenza viral RNA, but it also interacts with influenza NS1 protein to restrict nuclear export of viral mRNA and viral replication. Nevertheless, our results demonstrated that hnRNP A2/B1 could interact with not only the NP of the WSN virus, but also the NP of seasonal H1N1 from 2002 or avian H6N1, suggesting that the NP-hnRNP A2/B1 interaction might not be strain-specific, and that hnRNP A2/B1 could be a target for developing antivirals against influenza A virus.

## Supporting information

S1 Fig hnRNP A1 interacts with NP and regulates the RNP activity of influenza A virus. **(A)** 293T cells were transfected with a plasmid that expresses FLAG-tagged WSN NP encoded or an empty vector. The lysates of transfected cells were subjected to anti-FLAG immunoprecipitation. **(B)** HeLa cells were transfected with siRNA against hnRNP A1, and then co-transfected with plasmids that express NP, PA, PB1, PB2, renilla luciferase, and the vRNA-like firefly luciferase reporter as described previously. At 24 h post-transfection, lysates from the transfected cells were collected and analyzed with the Dual-Luciferase Reporter Assay and by immunoblotting with anti-hnRNP A2/B1 and anti-β actin antibodies. *** *P* < 0.005.
(TIFF)

S1 Table The sequences of primers used for RT and real-time PCR in this study. (DOCX)
