# Supplementary figures and images for: Cellular hnRNP A2/B1 interacts with the NP of influenza A virus and impacts viral replication
Source: PLoS One. 2017 Nov 16;12(11):e0188214. doi: 10.1371/journal.pone.0188214 (PMC5690641; doi:10.1371/journal.pone.0188214)

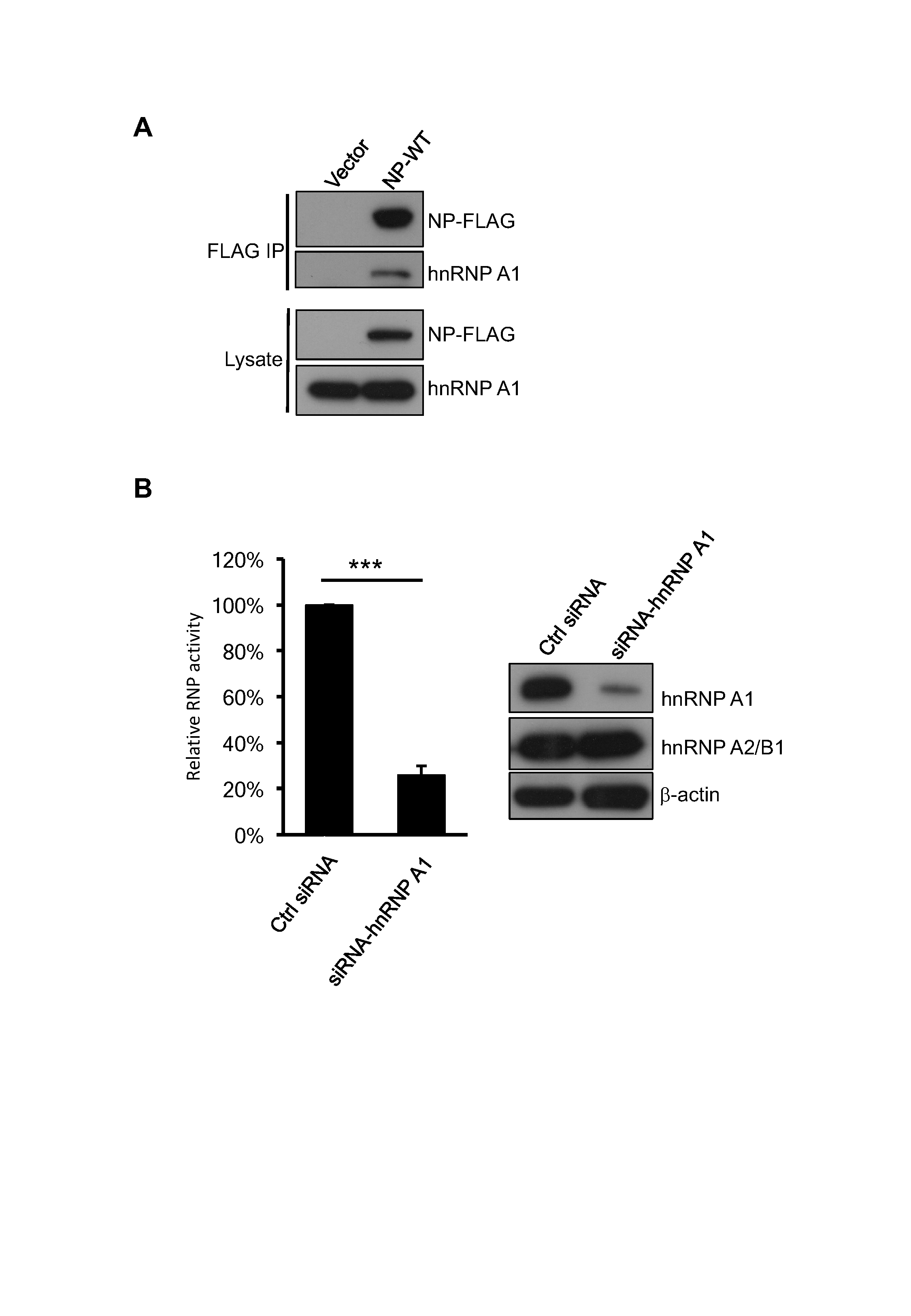

Supplement: S1 Fig — (A) 293T cells were transfected with a plasmid that expresses FLAG-tagged WSN NP encoded or an empty vector. The lysates of transfected cells were subjected to anti-FLAG immunoprecipitation. (B) HeLa cells were transfected with siRNA against hnRNP A1, and then co-transfected with plasmids that express NP, PA, PB1, PB2, renilla luciferase, and the vRNA-like firefly luciferase reporter as described previously. At 24 h post-transfection, lysates from the transfected cells were collected and analyzed with the Dual-Luciferase Reporter Assay and by immunoblotting with anti-hnRNP A2/B1 and anti-β actin antibodies. *** P < 0.005. (TIFF) [file pone.0188214.s001.tiff]
