# Supplementary material for: Cellular hnRNP A2/B1 interacts with the NP of influenza A virus and impacts viral replication
Source: PLoS One. 2017 Nov 16;12(11):e0188214. doi: 10.1371/journal.pone.0188214 (PMC5690641; doi:10.1371/journal.pone.0188214)
Supplement: S1 Table — (DOCX) [file pone.0188214.s002.docx]

**S1 Table**. The sequences of primers used for RT and real-time PCR in this study.

| Primer name | Sequence (5’ to 3’) |
| --- | --- |
| Firefly luciferase vRNA-RT | GACGTCATGAATAGGATGAATCGAGCAAAAGCAGGGTGACAAAG |
| Firefly luciferase mRNA-RT | CGCAGATCGTTCGAGTCGTTTTTTTTTTTTTTTTTTTTTTATCATTAC |
| Renilla luciferase mRNA-RT | CGCAGATCGTTCGAGTCGTTTCATCAGGTGCATCTTCTTGC |
| Firefly luciferase vRNA-F | GACGTCATGAATAGGATGAATCGAGCAAAAGCAGGGTGACAAAG |
| Firefly luciferase vRNA-R | CATTACACGGCGATCTTTCC |
| Firefly luciferase mRNA-F | CGCAGATCGTTCGAGTCG |
| Firefly luciferase mRNA-R | CGACGCAAGAAAAATCAGAGAGA |
| Renilla luciferase mRNA-F | CGCAGATCGTTCGAGTCG |
| Renilla luciferase mRNA-R primer | CGCAGATCGTTCGAGTCGT |
| human β-actin-F | CATGTACGTTGCTATCCAGGC |
| human β-actin-R | CTCCTTAATGTCACGCACGAT |
| RT: reverse transcription; F: forward; R: reverse | |
